# Addition of Rituximab in Reduced Intensity Conditioning Regimens for B-Cell Malignancies Does Not Influence Transplant Outcomes: EBMT Registry Analyses Following Allogeneic Stem Cell Transplantation for B-Cell Malignancies

**DOI:** 10.3389/fimmu.2020.613954

**Published:** 2021-02-02

**Authors:** Agnieszka Tomaszewska, Madan Jagasia, Eric Beohou, Steffie van der Werf, Didier Blaise, Edward Kanfer, Noel Milpied, Péter Reményi, Fabio Ciceri, Jean H. Bourhis, Patrice Chevallier, Carlos Solano, Gerard Socié, Benedetto Bruno, Alessandro Rambaldi, Luca Castagna, Nicolaus Kröger, Paolo Corradini, Boris Afanasyev, Marco Ladetto, Dietger Niederwieser, Christof Scheid, Henrik Sengeloev, Frank Kroschinsky, Ibrahim Yakoub-Agha, Helene Schoemans, Christian Koenecke, Olaf Penack, Zinaida Perić, Hildegard Greinix, Rafael F. Duarte, Grzegorz W. Basak

**Affiliations:** ^1^ Department of Hematology, Transplantology and Internal Medicine, Medical University of Warsaw, Warsaw, Poland; ^2^ Division of Hematology-Oncology, Vanderbilt University Medical Center, Nashville, TN, United States; ^3^ EBMT Paris Statistical Unit, Paris, France; ^4^ EBMT Data Office, Leiden, Netherlands; ^5^ Institut Paoli Calmettes, Marseille, France; ^6^ Hammersmith Hospital, Imperial College Healthcare, London, United Kingdom; ^7^ Hopital Haut-leveque, Bordeaux, France; ^8^ Dél-pesti Centrumkórház, Budapest, Hungary; ^9^ Ospedale San Raffaele s.r.l., Milan, Italy; ^10^ Gustave Roussy Institute de Cancérologie, Val de Marne, France; ^11^ CHU Nantes, Nantes, France; ^12^ Hospital Clínico Universitario, Valencia, Spain; ^13^ Hopital St. Louis, Paris, France; ^14^ A.O.U. Citta della Salute e della Scienza di Torino, Universita di Torino, Turin, Italy; ^15^ Azienda Ospedaliera Papa Giovanni XXIII, Bergamo, Italy; ^16^ Istituto Clinico Humanitas, Milan, Italy; ^17^ University Hospital Eppendorf, Hamburg, Germany; ^18^ Fondazione IRCCS Istituto Nazionale dei Tumori, University of Milano, Milan, Italy; ^19^ First State Pavlov Medical University of St. Petersburg, St. Petersburg, Russia; ^20^ H SS. Antonio e Biagio, Alessandria, Italy; ^21^ University Hospital Leipzig, Leipzig, Germany; ^22^ University of Cologne, Cologne, Germany; ^23^ Rigshospitalet, Copenhagen, Denmark; ^24^ Universitätsklinikum Dresden, Dresden, Germany; ^25^ Hôpital Huriez CHRU, Lille, France; ^26^ University Hospitals Leuven and KU Leuven, Leuven, Belgium; ^27^ Hannover Medical School, Hannover, Germany; ^28^ Department of Hematology, Oncology, and Tumor Immunology, Charité – Universitätsmedizin Berlin, Berlin, Germany; ^29^ University Hospital Center Rebro, Zagreb, Croatia; ^30^ Department of Hematology and Oncology, Medical University of Graz, Graz, Austria; ^31^ Hospital Universitario Puerta de Hierro Majadahonda, Madrid, Spain

**Keywords:** transplantation, B-cell malignancy, conditioning, rituximab, non-relapse mortality after hematopoietic cell transplantation, graft-versus-host disease

## Abstract

Rituximab (R) is increasingly incorporated in reduced intensity conditioning (RIC) regimens for allogeneic hematopoietic cell transplantation (alloHCT) in patients with B-cell malignancies, not only to improve disease control, but also to prevent graft-versus-host disease (GVHD). There are no randomized prospective data to validate this practice, although single center data and the CIBMTR analysis have shown promising results. We aimed at validation of these findings in a large registry study. We conducted a retrospective analysis using the EBMT registry of 3,803 adult patients with B-cell malignancies undergoing alloHCT (2001–2013) with either rituximab (R-RIC-9%) or non-rituximab (RIC-91%) reduced intensity regimens respectively. Median age and median follow up were 55 years (range 19.1–77.3) and 43.2 months (range 0.3–179.8), respectively. There was no difference in transplant outcomes (R-RIC vs RIC), including 1-year overall survival (69.9% vs 70.7%), 1-year disease-free survival (64.4% vs 62.2%), 1-year non-relapse mortality (21% vs 22%), and day-100 incidence of acute GVHD 2-4° (12% vs 12%). In summary, we found that addition of rituximab in RIC regimens for B-cell malignancies had no significant impact on major transplant outcome variables. Of note, data on chronic GVHD was not available, limiting the conclusions that can be drawn from the present study.

## Introduction

An optimal outcome after allogeneic hematopoietic cell transplantation (alloHCT), includes not only cure of the underlying malignancy, but minimizing the incidence of GVHD, both acute and chronic. Reduced intensity conditioning (RIC) allows patients in higher age groups and/or with significant comorbidities to access alloHCT, while minimizing transplant related mortality, and preserving graft-versus-tumor effect (1, 2).

RIC regimens have historically been designed to be unrelated to underlying disease, but at the same time, choice of agents used often has a scientific rationale for offering tumor-specific disease control (3, 4).

RIC regimens containing rituximab, an anti-CD20 antibody with anti-neoplastic activity, have been included for targeting B-cell malignancies and single center studies suggest a superior alloHCT outcome compared to center-specific historical cohorts (4–6). Recent data from the Center of International Blood and Marrow Transplant Registry (CIBMTR) suggests that the effect of rituximab may be confined to some subsets of patients or when specific preparative regimens are used (7). We studied the effect of rituximab using the European Society for Blood and Marrow Transplantation (EBMT) registry to attempt to cross validate the above mentioned findings.

## Methods

### Patient Selection and Treatment Plan

We analyzed 3,803 patients with B cell lymphoid malignancies (FL – follicular lymphoma, DLBCL – diffuse large B cell lymphoma, MCL – mantle cell lymphoma, CLL – chronic lymphocytic leukemia, SLL – small lymphocytic lymphoma), who received a RIC allo-HCT between 2001 and 2013 and were reported to the registry of the EBMT. Patients undergoing myeloablative HCT (total body irradiation > 6 Gy, or busulfan > 9 mg/kg) or alemtuzumab containing regimens were excluded. Umbilical cord and haploidentical HCT were excluded. All RIC or R-RIC regimens were included and were stratified as fludarabine (Flu), busulfan (Bu2), other FluBu regimens, other Flu containing regimens or other regimens.

Patients undergoing alloHCT often receive anti-T-cell globulin (ATG) as a part of the conditioning regimen for GVHD prophylaxis. Thus, regimens were stratified for ATG or non-ATG regimens, within each group of RIC or R-RIC, for analyses. Details of ATG dosing and schedule were not available. Similarly, the details of the dosing and schedule of rituximab were not available in the EBMT registry. GVHD prophylaxis details were captured as cyclosporine (CSA), mycophenolate mofetil (MMF), CSA plus MMF, CSA plus methotrexate (MTX), or other regimens.

Baseline demographics including follow-up, age at transplant, gender, diagnosis, disease status at alloHCT, year of alloHCT, donor type, recipient-donor gender match or mismatch, source of stem cells were obtained from the registry.

Maximum acute GVHD grade within the first 100 days was available in the majority of patients. In contrast, chronic GVHD status was available in a small minority of the patients and was not analyzed. Transplant outcomes of overall survival (OS), relapse incidence, and non-relapse mortality were available.

### Statistical Analysis

Comparison of patient and transplant characteristics was conducted using the Mann-Whitney test for continuous variables and the chi-squared test for categorical variables. Overall survival, progression-free survival, and GVHD-free/relapse-free survival (GRFS) were estimated using the Kaplan-Meier method. Multivariable prediction models for continuous and binary outcomes were made using standard and logistic regression, respectively. Based on comparison of patient and transplant characteristics, age at HCT, year of HCT, preparative regimen, donor type, and GVHD prophylaxis were included in the multivariate analyses. Cumulative incidence functions were calculated using Gray’s Method (8). Cumulative incidence of relapse, and non-relapse mortality, were calculated. *P* values less than 0.05 were considered statistically significant. All statistical analyses were performed using R software by the EBMT statistical team.

## Results

### Patient Characteristics

**Table 1** outlines patient and transplant characteristics. The overall cohort consisted of 3,803 patients (R-RIC: 350; RIC: 3,453) with a median follow up of 43.2 months (range, 0.3–179.8). Patients receiving R-RIC had a lower median age (54.2 y; range, 19.8–74.1) compared to RIC (55 y; range, 19.1–77.3) (p=0.01), and underwent transplant more recently (2001–2007: 35.2% RIC and 19.1% R-RIC, 2008–2013: 64.8% RIC, 80.9% R-RIC; median year of HCT: R-RIC vs. RIC; 2010 vs. 2009, p<0.0001). GVHD prophylaxis regimens were significantly different with higher incidence of CSA plus MMF in the RIC group and a higher incidence of CSA plus MTX in the R-RIC group. Preparative regimens varied significantly (p<0.0001) with fludarabine plus cyclophosphamide being more commonly used in the R-RIC group (46.3%) compared to the RIC group (23.9%).

**Table 1:** Pre-transplant and transplant characteristics.

Variable	Overall (N = 3803)	NO Rituximab (N = 3453)	RITUXIMAB (N = 350)	P
Follow up for survivors (months), median (min-max)	43.2 (0.3–179.8)	42.7 (0.3–179.8)	44.6 (1.5–126.8)	0.82
Age of patient at HCT (in years), median (min-max)	55.0 (19.1–77.3)	55.0 (19.1–77.3)	54.2 (19.8–74.1)	0.01
Age of patient at HCT (categorical), n (%)				0.07
18**–**49 y	1166 (30.7)	1043 (30.2)	123 (35.1)	
+50 y	2637 (69.3)	2410 (69.8)	227 (64.9)	
Gender of patient, n (%)				0.89
Male	2601 (68.4)	2360 (68.3)	241 (68.9)	
Female	1202 (31.6)	1093 (31.7)	109 (31.1)	
Diagnosis, n (%)				0.82
FL	1108 (29.2)	1005 (29.1)	103 (29.4)	
DLBCL	657 (17.3)	592 (17.2)	65 (18.6)	
MCL	655 (17.2)	592 (17.2)	63 (18.0)	
CLL/SLL B-cell	517 (13.6)	476 (13.8)	41 (11.7)	
Unspecified CLL	863 (22.7)	785 (22.8)	78 (22.3)	
Missing	3	3	0	
Disease status at HCT, n (%)				0.48
PR/nPR	1157 (33.3)	1062 (33.6)	95 (30.2)	
Relapse/progression	810 (23.3)	741 (23.5)	69 (21.9)	
CR/nCR	1369 (39.4)	1232 (39.0)	137 (43.5)	
Primary refractory/no CR	108 (3.1)	96 (3.0)	12 (3.8)	
Other	28 (0.8)	26 (0.8)	2 (0.6)	
Missing	331	296	35	
Year of HCT, median (min-max)	2009.0 (2001.0–2013.0)	2009.0 (2001.0–2013.0)	2010.0 (2002.0–2013.0)	<0.0001
Year of HCT (categorical), n (%)				<0.0001
2001**–**2007	1284 (33.8)	1217 (35.2)	67 (19.1)	
2008**–**2013	2519 (66.2)	2236 (64.8)	283 (80.9)	
Time from diagnosis to HCT (months), median (min-max)	48.5 (0.3–665.3)	48.7 (0.3–665.3)	47.2 (4.2–244.5)	0.62
Donor type, n (%)				0.04
Related	1995 (52.5)	1793 (51.9)	202 (57.7)	
Unrelated	1808 (47.5)	1660 (48.1)	148 (42.3)	
Sex mismatch, n (%)				0.42
Other	2881 (76.4)	2620 (76.6)	261 (74.6)	
Female to male	888 (23.6)	799 (23.4)	89 (25.4)	
Missing	34	34	0	
Stem cell sources, n (%)				0.18
BM	271 (7.2)	253 (7.3)	18 (5.2)	
PB	3518 (92.8)	3191 (92.7)	327 (94.8)	
Missing	14	9	5	
GVHD prevention, n (%)				<0.0001
CSA	498 (13.9)	463 (14.3)	35 (10.3)	
MMF	274 (7.6)	261 (8.0)	13 (3.8)	
CSA+ MMF	1436 (40.1)	1353 (41.7)	83 (24.4)	
CSA + MTX	1166 (32.5)	1010 (31.1)	156 (45.9)	
Other	210 (5.9)	157 (4.8)	53 (15.6)	
Missing	219	209	10	
aGVHD, n (%)				0.18
No	2524 (68.0)	2278 (67.6)	246 (71.3)	
Yes	1190 (32.0)	1091 (32.4)	99 (28.7)	
Missing	89	84	5	
Preparative regimens, n (%)				<0.0001
FluBu2	714 (19.0)	672 (19.7)	42 (12.0)	
Flu-based (+/-others)	1119 (29.7)	1031 (30.2)	88 (25.1)	
FluCy	977 (26.0)	815 (23.9)	162 (46.3)	
FluMel	724 (19.2)	675 (19.8)	49 (14.0)	
Others	230 (6.1)	221 (6.5)	9 (2.6)	
Missing	39	39	0	
ATG used, n (%)				0.12
No	2621 (69.6)	2364 (69.2)	257 (73.4)	
Yes	1143 (30.4)	1050 (30.8)	93 (26.6)	
Missing	39	39	0	

HCT, hematopoietic cell transplantation; FL, follicular lymphoma; DLBCL, diffuse large B cell lymphoma; MCL, mantle cell lymphoma; CLL, chronic lymphocytic leukemia; SLL, small lymphocytic lymphoma; PR/nPR, partial remission/near partial remission; CR/nCR, complete remission/near complete remission; BM, bone marrow; PB, peripheral blood; CSA, cyclosporine A; MMF, mycophenolate mofetil; MTX, methotrexate; GvHD, graft versus host disease; aGvHD, acute graft versus host disease; Flu, fludarabine; Bu, busulfan; Cy, cyclophosphamide; Mel, melphalan; ATG, antithymocyte globulin (anti T-cell globulin).

### Survival, Relapse, Progression-Free Survival, Non-Relapse Mortality and 100-Day Cumulative Incidence of Acute GVHD

**Figure 1:**
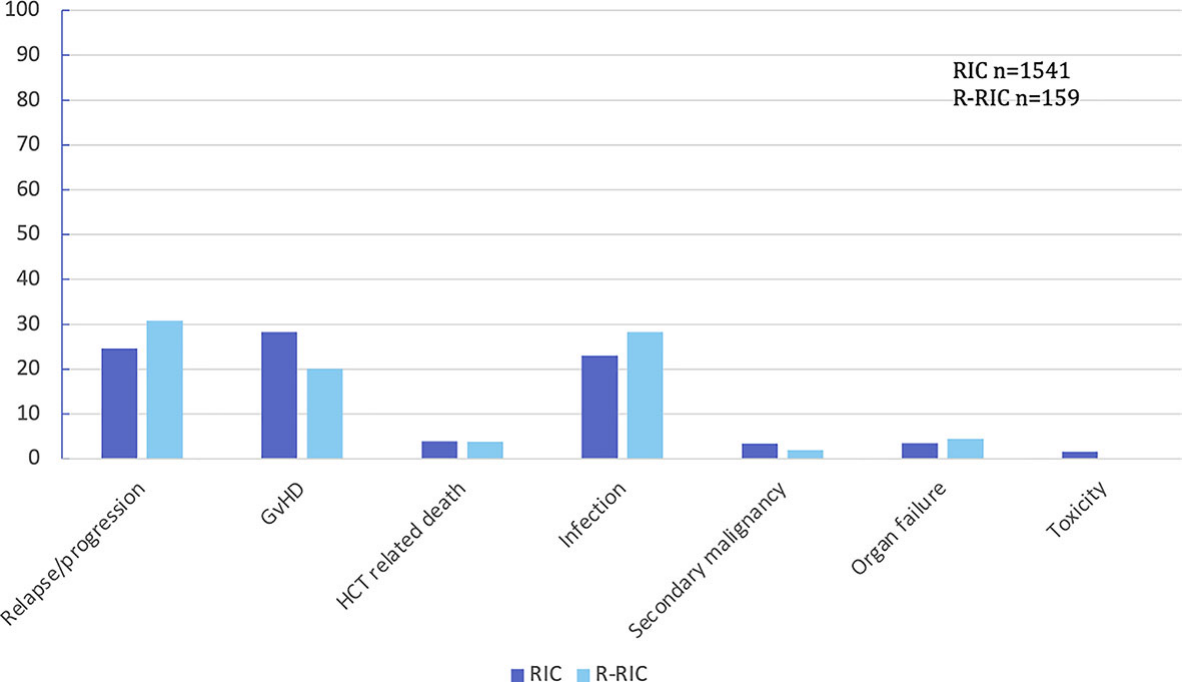
Main causes (percentages) of death in patients’ cohorts: RIC vs R-RIC. GvHD, graft versus host disease; HCT, hematopoietic cell transplantation.

**Table 2** outlines the above endpoints. There were no significant differences in the 1-year OS, 1-year relapse rate, 1-year non-relapse mortality, 1-year disease free survival, and the grade 2–4 aGvHD incidence between analysed cohorts of patients as well as there were no differing causes of death (**Figure 1** in the manuscript and **Table 3S** in supplementary materials). Chronic GVHD analyses were not undertaken given the extent of missing data, and lack of data in the context of the NIH classification system.

**Table 2:** Transplant outcomes for entire cohort, patients excluding those receiving fludarabine plus busulfan, and patients excluding those receiving anti-T-cell globulin.

Outcome	STRATA	ALL PATIENTS		FLUBU2 EXCLUDED		ATG EXCLUDED	
		% (95% CI)	P	% (95% CI)	P	% (95% CI)	P
1 Y RELAPSE INCIDENCE	RIC	15 (14–16)	0.37	14 (13–16)	0.34	15 (13–16)	0.60
	R-RIC	13 (10–17)		12 (9–16)		13 (9–17)	
1 Y NON-RELAPSE MORTALITY	RIC	22 (20–23)	0.62	22 (21–24)	0.56	22 (20–23)	0.63
	R-RIC	21 (17–26)		22 (18–27)		21 (16–26)	
1 Y DISEASE FREE SURVIVAL	RIC	62.2 (60.6–63.9)	0.78	62.1 (60.2–64)	0.79	62.5 (60.6–64.6)	0.98
	R-RIC	64.4 (59.5–69.7)		63.7 (58.5–69.5)		64.8 (59.1–71)	
1 Y OVERALL SURVIVAL	RIC	70.7 (69.2–72.3)	0.81	70 (68.2–71.8)	0.79	70.9 (69.1–72.8)	0.72
	R-RIC	69.9 (65.2–75)		69 (64–74.5)		70.8 (65.3–76.6)	
100 D aGVHD GRADE 2**–**4	RIC	12 (11–14)	0.64	15 (14–16)	0.12	15 (14–17)	0.77
	R-RIC	12 (9–16)		12 (10–13)		11 (10–13)	

AGVHD, acute graft versus host disease; Flu, fludarabine; Bu, busulfan; ATG, antithymocyte globulin (anti T-cell globulin).

### Subset Analyses

Given the differential impact of preparative regimens on outcomes as reported by the CIBMTR (7), we studied two subsets (all patients excluding those receiving fludarabine plus busulfan [FluBu2 excluded]; and all patients excluding those receiving ATG [ATG excluded]). The percent of patients in these various subsets and use of GVHD prophylaxis in these subsets are outlined in supplementary materials **Table 1S** and **2S**, respectively. Contrary to the findings in the CIBMTR cohort, there were no differences in the outcome endpoints in multivariable analyses, adjusted for age, year of HCT, GVHD prophylaxis and donor type (**Table 3**).

**Table 3:** Transplant outcomes: stratified by patients subsets.

OUTCOMES	ALL PATIENTS^		FLU-BU2 EXCLUDED^		ATG EXCLUDED^	
	HR (95% CI) RITUXIMAB VS NO RITUXIMAB	P	HR (95% CI) RITUXIMAB VS NO RITUXIMAB	P	HR (95% CI) RITUXIMAB VS NO RITUXIMAB	P
RELAPSE INCIDENCE	0.93 (0.71–1.21)	0.57	0.88 (0.66–1.18)	0.39	0.94 (0.68–1.29)	0.69
NON-RELAPSE MORTALITY	1.07 (0.85–1.33)	0.58	1.09 (0.86–1.39)	0.46	1.09 (0.84–1.42)	0.51
DISEASE FREE SURVIVAL	1.01 (0.85–1.19)	0.94	1.01 (0.84–1.21)	0.91	1.03 (0.85–1.26)	0.74
OVERALL SURVIVAL	1.06 (0.88–1.26)	0.55	1.08 (0.89–1.31)	0.45	1.08 (0.88–1.34)	0.46
ACUTE GVHD 3**–**4**°**	1.09 (0.79–1.51)	0.60	1.1 (0.78–1.56)	0.59	1.09 (0.75–1.58)	0.66

^Adjusted for age at transplant, year of transplant, preparative regimen, GVHD prophylaxis and donor type.

Flu, fludarabine; Bu, busulfan; ATG, anti T-cell globulin; D-donor, R-recipient; GVHD, graft versus host disease.

## Discussion

Historically, preparative regimens have remained unrelated to the underlying disease (1–3). The goal of the regimen is to achieve adequate immunoablation to facilitate donor cell engraftment, while maintaining adequate control of the underlying disease (1, 2). Although, there are a multitude of regimens in common practice, there is little prospective data that regimens truly affect HCT outcomes in patients with B-cell NHL. Clinical observations of reduced GVHD in patients having received rituximab in chemotherapy regimens preceding HCT were noted prior to the elucidation of B-cell biology and chronic GVHD (4, 5). Rituximab treatment within six months prior to RIC allo-HCT for any hematologic malignancy has been shown to reduce the incidence of extensive cGVHD from 45.8% to 20.1% (9).

In this study, we report that the addition of rituximab to RIC regimen had no incremental impact on outcomes of alloHCT in patients with B-cell malignancies. This study is in contrast to the CIBMTR analyses showing that the addition of rituximab was associated with improved PFS (7). It is interesting to note that although a PFS advantage was seen in the entire CIBMTR cohort, the survival benefit was confined only to patients not receiving fludarabine plus busulfan preparative regimen and with a higher cumulative dose of rituximab (7). In presented study the cohort of patients was larger than in the CIBMTR study (3,803 vs 1,401) but the prevalence of rituximab in preparative regimens for B-cell malignancies in our study was approximately 10% in contrast to 27% in the CIBMTR one.

In a separate CIBMTR study confined only to patients with follicular lymphoma, comparing the two most commonly used RIC approaches – fludarabine and busulfan (FluBu) versus fludarabine, cyclophosphamide, and rituximab (FCR) reported survival outcomes (OS, PFS, NRM) were not significantly different (10). The only noted benefit in this study was a decrease in incidence of chronic GVHD with R-RIC compared to RIC regimens (10). Data from the retrospective study of Kennedy et al. showed that use of RIC with FCR was associated with decreased chronic GVHD and improved OS (6).

In our study, data on chronic GVHD incidence and severity in the context of the NIH classification system was not available, limiting the conclusions that can be drawn.

Rituximab was used in the treatment of chronic GVHD and studied as a pre-emptive strategy in post-HCT setting for prevention of chronic GVHD (9, 11–13). It is an established second-line agent in the treatment of steroid-refractory cGVHD (12–16), and has been used in conjunction with corticosteroids as initial cGVHD therapy with moderate success (17). There is increasing evidence that B cells play a role in cGVHD pathogenesis (18–20), and it has been hypothesized that reducing B cell alloimmunity may decrease cGVHD incidence (16). Alloreactive antibodies against H-Y antigens are strongly associated with the occurrence of cGVHD (21, 22), and post-transplant rituximab has been shown to decrease allogeneic H-Y antibody development (16).

The above data may suggest that the effect of rituximab in modulating outcome is modest at the best and confined to regimens which are inherently associated with a slower rate of converting to full donor chimerism (non-busulfan containing regimens) (6, 7, 10). The survival benefit was noted in the high dose rituximab group (7).

Finally, what is the role of anti-thymocyte globulin in rituximab containing preparative regimens? The cumulative incidence of grade 3–4 acute GVHD, relapse, non-relapse mortality, disease-free, and overall survival did not show any significant differences in patients who did not receive ATG in the preparative regimen.

However, it does not answer the question if ATG can be safely omitted without compromising outcomes when rituximab containing preparative regimens are used for unrelated donor transplants. As preparative regimens are often “bundled” together with certain GVHD prophylaxis, it is challenging to study the differential impact of the regimen versus the GVHD prophylaxis. In our study, given the large sample size, we could adjust for the GVHD prophylaxis and did not see any interaction.

In conclusion, we could not demonstrate the benefit of adding rituximab to RIC regimens for B-cell malignancies on HCT outcomes. Although our study was limited by the absence of chronic GVHD data, the larger sample size in contrast to the positive the CIBMTR study, suggests that the effect of rituximab needs to be systematically re-examined. Many centers have adopted this institutional practice, despite the lack of well conducted mechanistic studies to optimize the dosage and schedule. The cost of rituximab is not trivial, and the transplant community needs to take a step back to re-examine the role of B-cell inhibitors in the preparative regimens. It is unlikely that a phase 3 study will ever be done to address this question. However, with the advent of more targeted B-cell receptor or signaling pathway inhibitors it is critical, that the transplant community should follow the conventional pathway of drug development with carefully conducted phase I studies, prior to accepting agents approved for other indications and integrating it into the preparative regimens.

## Data Availability Statement

The original contributions presented in the study are included in the article/**Supplementary Material**. Further inquiries can be directed to the corresponding author.

## Author Contributions

AT and MJ contributed equally to writing this manuscript. EB analyzed the data. All authors contributed to the article and approved the submitted version.

## Conflict of Interest

The authors declare that the research was conducted in the absence of any commercial or financial relationships that could be construed as a potential conflict of interest.
