# Supplementary material for: Addition of Rituximab in Reduced Intensity Conditioning Regimens for B-Cell Malignancies Does Not Influence Transplant Outcomes: EBMT Registry Analyses Following Allogeneic Stem Cell Transplantation for B-Cell Malignancies
Source: Front Immunol. 2021 Feb 2;11:613954. doi: 10.3389/fimmu.2020.613954 (PMC7884746; doi:10.3389/fimmu.2020.613954)
Supplement: Supplementary file 1 [file DataSheet_1.zip › Supplementary Table 1.DOCX]

Table 1S. Subsets of patients receiving R-RIC vs RIC: stratified by those not receiving fludarabine plus busulfan or anti-T-cell globulin.

|  | No Rituximab  (% of total) | Rituximab based  (% of total) | Total |
| --- | --- | --- | --- |
| All regimens | 3453  (90.8%) | 350  (9.2%) | 3803 |
| Flu-Bu2 Excluded | 2742  (89.9%) | 308  (10.1%) | 3050 |
| ATG Excluded | 2364  (90.2%) | 257  (9.8%) | 2621 |

Abbreviations: Flu - fludarabine, Bu - busulfan, ATG – antithymocyte globulin (anti T-cell globulin).
