# Supplementary material for: Addition of Rituximab in Reduced Intensity Conditioning Regimens for B-Cell Malignancies Does Not Influence Transplant Outcomes: EBMT Registry Analyses Following Allogeneic Stem Cell Transplantation for B-Cell Malignancies
Source: Front Immunol. 2021 Feb 2;11:613954. doi: 10.3389/fimmu.2020.613954 (PMC7884746; doi:10.3389/fimmu.2020.613954)
Supplement: Supplementary file 1 [file DataSheet_1.zip › Supplementary Table 2.DOCX]

Table 2S. Acute GVHD prophylaxis: stratified by use of preparative regimens

| VARIABLE | OVERALL  (N=3803) | CSA  (N=498) | MMF  (n=274) | CSA + MMF  (N=1436) | CSA + MTX  (N=1166) | OTHER  (N=210) | | P |
| --- | --- | --- | --- | --- | --- | --- | --- | --- |
| REGIMENS, N (%) |  |  |  |  |  | |  | <0.0001 |
| FLU-BU2 | 692 (19.5) | 217 (43.8) | 24 (8.8) | 186 (13.1) | 236 (20.4) | 29 (13.9) | |  |
| FLU BASED (+/- OTHERS) | 1056 (29.7) | 53 (10.7) | 171 (62.6) | 703 (49.6) | 99 (8.5) | 30 (14.4) | |  |
| FLU-CY | 921 (25.9) | 137 (27.7) | 36 (13.2) | 285 (20.1) | 392 (33.8) | 71 (34) | |  |
| FLU-MEL | 682 (19.2) | 61 (12.3) | 26 (9.5) | 200 (14.1) | 321 (27.7) | 74 (35.4) | |  |
| OTHERS | 202 (5.7) | 27 (5.5) | 16 (5.9) | 43 (3.0) | 111 (9.6) | 5 (2.4) | |  |
| MISSING | 31 | 3 | 1 | 19 | 7 | 1 | |  |

Abbreviations: CSA – ciclosporine A, MMF – mycophenolate mofetil, MTX - methotrexate, Flu - fludarabine, Bu - busulfan, Cy - cyclophosphamide, Mel – melphalan
