# Supplementary material for: Addition of Rituximab in Reduced Intensity Conditioning Regimens for B-Cell Malignancies Does Not Influence Transplant Outcomes: EBMT Registry Analyses Following Allogeneic Stem Cell Transplantation for B-Cell Malignancies
Source: Front Immunol. 2021 Feb 2;11:613954. doi: 10.3389/fimmu.2020.613954 (PMC7884746; doi:10.3389/fimmu.2020.613954)
Supplement: Supplementary file 1 [file DataSheet_1.zip › Supplementary Table 3.DOCX]

Table 3S. Cause of death: stratified by R-RIC vs RIC.

| VARIABLE | OVERALL  (N=1700) | NO RITUXIMAB  (N=1541) | RITUXIMAB  (N=159) |
| --- | --- | --- | --- |
| Cause of death, n (%) |  |  |  |
| Relapse/progression | 428 (25.2) | 379 (24.6) | 49 (30.8) |
| Secondary malignancy/PTLD | 56 (3.3) | 53 (3.4) | 3 (1.9) |
| GVHD | 468 (27.5) | 436 (28.3) | 32 (20.1) |
| Infection | 399 (23.5) | 354 (23.0) | 45 (28.3) |
| Organ damage/failure | 61 (3.6) | 54 (3.5) | 7 (4.4) |
| Toxicity | 25 (1.5) | 25 (1.6) | 0 (0.0) |
| HCT-related death | 66 (3.9) | 60 (3.9) | 6 (3.8) |
| Missing/unknown | 197 (11.6) | 180 (11.7) | 17 (10.7) |

Abbreviations: PTLD – post-transplant lymphoproliferative disease; GVHD – graft versus host disease
